# Long-term Cyclability of Substoichiometric Silicon Nitride Thin Film Anodes for Li-ion Batteries

**DOI:** 10.1038/s41598-017-13699-0

**Published:** 2017-10-17

**Authors:** Asbjørn Ulvestad, Hanne Flåten Andersen, Jan Petter Mæhlen, Øystein Prytz, Martin Kirkengen

**Affiliations:** 1 Department of Energy Systems, Institute for Energy Technology, P. O. Box 40, NO-2027 Kjeller, Norway; 2 Department of Physics, Centre for Materials Science and Nanotechnology, University of Oslo, P. O. Box 1048 Blindern, NO-0316 Oslo, Norway

## Abstract

Silicon has been the subject of an extensive research effort aimed at developing new anode materials for lithium ion batteries due to its large specific and volumetric capacity. However, commercial use is limited by a number of degradation problems, many of which are related to the large volume change the material undergoes during cycling in combination with limited lithium-diffusivity. Silicon rich silicon oxides (SiO_x_), which converts into active silicon and inactive lithium oxide during the initial lithiation, have attracted some attention as a possible solution to these issues. In this work we present an investigation of silicon rich amorphous silicon nitride (a-SiN_x_) as an alternative convertible anode material. Amorphous SiN_0.89_ thin films deposited by plasma enhanced chemical vapour deposition show reversible reactions with lithium when cycled between 0.05 and 1.0 V vs. Li^+^/Li. This material delivers a reversible capacity of approximately 1,200 mAh/g and exhibits excellent cycling stability, with 41 nm a-SiN_0.89_ thin film electrodes showing negligible capacity degradation over more than 2,400 cycles.

## Introduction

The development of the lithium ion battery has been one of the key elements in the revolution of portable electronic devices seen over the last decades. Increasing demands, particularly in the mobility sector, has led to an extensive effort to develop safer and cheaper electrode materials with higher energy density and better cycle life. The vast majority of current commercial lithium ion batteries use carbonaceous anodes, which have a rather limited theoretical capacity of 372 mAh/g^1^. Silicon has attracted much attention as a next generation anode material due to its very high theoretical capacity (3,579 mAh/g (Li_15_Si_4_)); however, there are a number of obstacles that must be overcome in order for it to become a viable commercial option. Several major issues are related to silicon undergoing a large volume change during lithiation and delithiation^2,3^. Uneven expansion and contraction caused by limited diffusion of lithium in the bulk can cause it to fracture, exposing new surface area as well as electronically disconnecting material from the electrode, rendering it inactive. Additionally, the large expansion and contraction of the surface can prevent the formation of a stable solid electrolyte interphase (SEI). Micro-scale silicon has also been reported to shows signs of migration during lithiation and delithiation^4^.

The most common method used to limit these effects is so called dimensional stabilization; reducing stresses by using nanometer-scale thin films, particles and nanorods, which themselves can be compact, porous or in a core-shell configuration^3,5–14^. Other methods include coatings^15–22^ and other surface modifications^23^, combination of silicon thin films with solid electrolytes^24^, and the use of silicon alloyed with other metals^24,25^. Another approach has been to use materials commonly referred to as *in-situ* formed alloys or convertible oxides^26^. First reported for the tin based composite oxide electrode by Fuji Photo Film Co.^27,28^, and later for silicon oxide (SiO) and mixtures of silicon oxide and tin oxide^29–31^, these materials are converted into a fine composite microstructure of a lithium alloy (e.g. Li_4.4_Sn or Li_15_Si_4_) and an inorganic lithium compound (e.g. Li_2_O) during the initial reduction cycle^32^; hence the name “*in-situ* formed alloy anodes”. In contrast to the conversion type electrodes, the initial conversion reaction of these materials is irreversible, and the inorganic lithium compound, hereinafter referred to as the matrix phase, is expected to be inactive; hence these materials tend to have high first cycle irreversible capacity. While ideally this should be avoided, it is the case for many high capacity anodes, emphasizing the need for the invention of a scalable and economic prelithiation method^33^. The matrix phase reduces the total volume change of the electrode during cycling and provides mechanical stabilization and lithium ion conductivity, and is thus expected to increase the electrode cycling stability; however, due to the added inactive mass, some reversible capacity is sacrificed relative to the pure metals in order to gain this stability. Since using anodes with higher capacity than about 1,200 mAh/g currently has a limited impact on the full cell capacity due to limitations of other components in the cell, this is usually regarded as an acceptable trade-off^34^. The electrochemical performance of these materials after conversion is otherwise expected to be similar to the resulting lithium alloys^32^.

The purpose of this work has been to investigate the performance of substoichiometric silicon nitride as an alternative *in-situ* formed alloy anode material for lithium ion batteries. Silicon nitride has been little investigated for this purpose, and the few previous publications on this topic have reported varying performance. On one extreme, nanoparticles and whiskers of Si_3_N_4_ have been used as an inactive buffer material for silicon nanoparticles^35^. On the other, Suzuki, *et al*.^36^ have reported a stable capacity of 1,300 mAh/g after 100 cycles for 200 nm SiN_0.92_ thin films. Ahn, *et al*.^37^ have reported on the performance of two 200 nm thin films with composition SiN_0.32_ and SiN_0.69_, the former having less than 80 mAh/g capacity for 645 cycles before suddenly increasing to 2,300 mAh/g for the next 200 cycles, while the latter stayed below 80 mAh/g during the entire cycling. Others have reported a reversible capacity of 40 mAh/g from particle based electrodes of stoichiometric Si_3_N_4_^38^. While the exact form of the conversion reaction has not been determined, silicon nitride has been proposed to convert into active silicon and inactive Si_3_N_4_ and/or Li_3_N^37,38^, or one of several lithium silicon nitride ternary phases during the initial lithiation^36^. This solid state conversion is expected to produce finely dispersed domains of the different phase constituents, and thus combine the high lithium storage capacity of silicon with the lithium ion conductivity and structural support of the other phases^37^. In this work we show that a-SiN_0.89_ thin films exhibit high reversible capacity of approximately 1,200 mAh/g and excellent cycling stability over more than 2,400 cycles.

## Results and Discussion

### Characterization of SiN_x_ films

**Figure 1:**
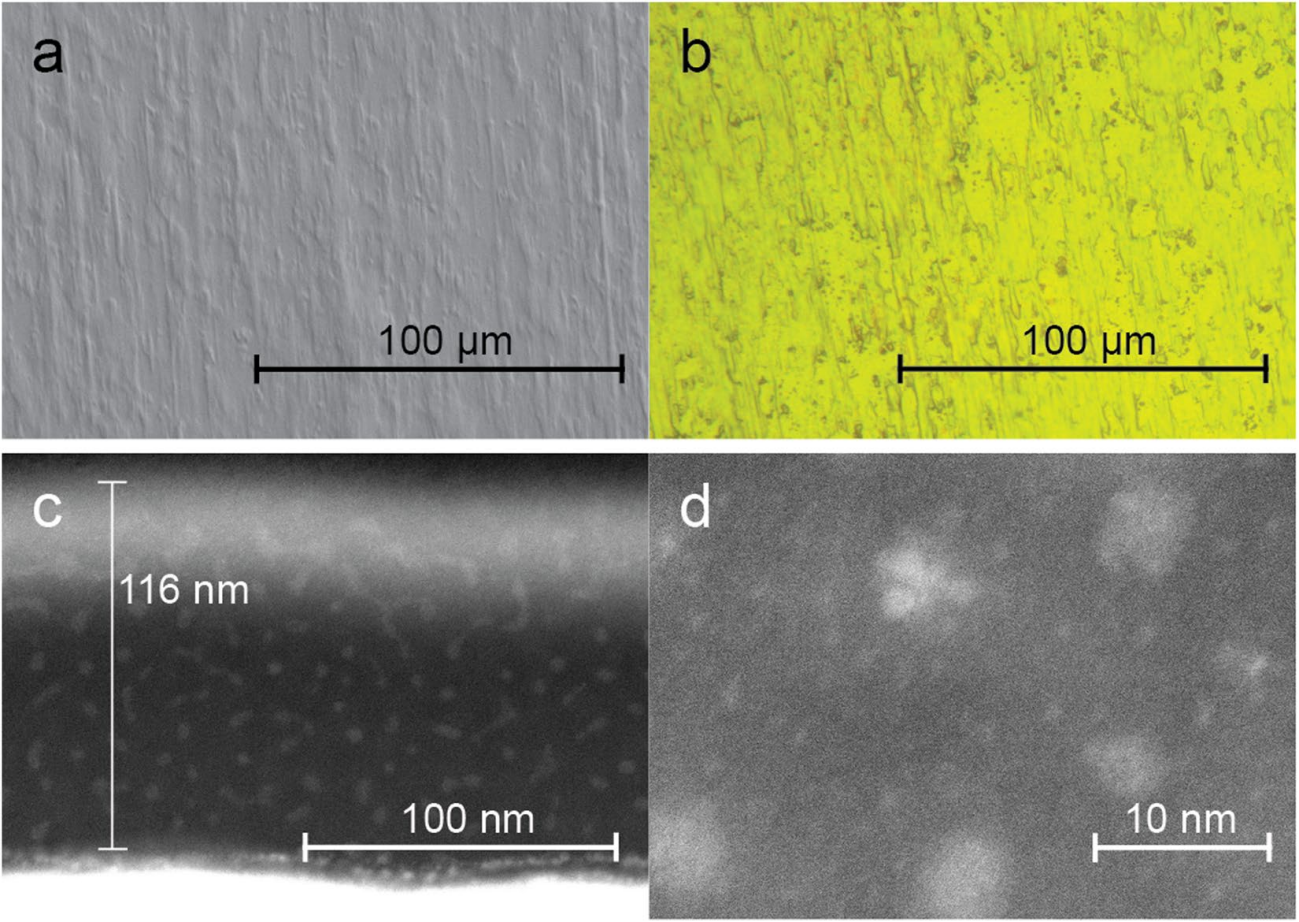
Optical, SEM and STEM micrographs of pristine a-SiN_0.89_ thin films. Plane view SEM (**a**) and optical (**b**) micrographs of the 156 nm a-SiN_0.89_ film. The structure of the surface is related to the structure of the rolled copper substrate. The uniform colour in the optical micrograph indicates a uniform film thickness. Cross section STEM HAADF images (**c** and **d**) of the film measured to be 114 nm using ellipsometry and 116 nm in STEM, showing the distribution and size of nanoscale inhomogeneities.

Amorphous SiN_x_ thin films were deposited using plasma enhanced chemical vapour deposition (PECVD) with silane (SiH_4_) and ammonia (NH_3_) as precursors (5:8 mixture by volume). Using ellipsometry, the film thicknesses were determined to be 41 nm, 80 nm, 114 nm, 156 nm, and 190 nm. Additionally, a pure silicon thin film was made as reference, with a thickness of 42 nm. The accuracy of the thickness measurements was verified for the 114 nm a-SiN_x_ film using scanning transmission electron microscopy (STEM). As seen in the high angle annular dark field (STEM-HAADF) image of the film cross section in Fig. 1c, the thickness of this film was measured to be 116 nm, which is within the experimental margin of error. Scanning electron microscopy (SEM) and optical microscopy analysis of the films, as seen for the 156 nm film in Fig. 1a and b, revealed no sign of island formation during deposition, and showed that the films covered the substrates evenly. The topography of the surface seen in these images is related to the structure of the rolled copper onto which the films are deposited. The film quality was also confirmed in the STEM analysis, which showed a dense film with a uniform thickness over the area which was analysed (~10 µm), despite the relative roughness of the substrate surface.

The average [N]/[Si] ratio of the films was determined from the measured refractive indices to be 1.00 (σ = 0.06) using equation (1). X-ray photoelectron spectroscopy (XPS) analysis of the 41 nm and 114 nm films, on the other hand, resulted in a [N]/[Si] ratio of 0.89 (σ = 0.03). The former method is dependent on an empirical relationship, and since the optical properties of PECVD deposited films greatly depend on deposition parameters, the latter is deemed most credible. Given that the films are expected to have the same composition, this is also supported by the smaller variation in the XPS measurements, and an average composition of SiN_0.89_ will therefore be used in the remainder of this work. The oxygen content of the a-SiN_x_ films was also determined in the XPS analysis to be 4.5 at.% (σ = 0.2 at.%). Given that the films were stored in ambient atmosphere; partial oxidation of the a-SiN_x_ is to be expected. The oxygen content of the pure silicon reference film was similarly determined to be 5.4 at.%. Material deposited by PECVD is also expected to have some hydrogen content, often up to several tens of atomic percent. Since hydrogen is not detectable by XPS, this was measured using secondary ion mass spectrometry (SIMS) analysis of the 114 nm thin film. Lack of suitable standards made it difficult to properly compensate for matrix effects, limiting the accuracy of this measurement; however, it did show that the hydrogen content was considerable, with an atomic concentration similar to that of silicon, in the range of approximately 20 to 30 at.%. While this constitutes only approximately 1–2% of the electrode mass, it may still affect the electrode performance. Given the large uncertainty, the hydrogen content is neglected when giving the atomic contents of other elements, unless otherwise stated.

Peak fitting of the Si 2p core level XPS spectra was performed using a procedure by Ingo, *et al*.^39^, in which the relative contributions of the Si-Si_4_, Si-Si_3_N, Si-Si_2_N_2_, Si-SiN_3_ and Si-N_4_ tetrahedra are separated. This revealed a significantly higher number of Si-Si_4_ tetrahedra than expected from a random mixing model, but that the material otherwise was well described by this model. This indicates that the a-SiN_x_ is not completely homogenous, and that some phase separation of pure silicon has occurred. The presence of inhomogeneities in the nanometer range was confirmed using STEM, which revealed clusters with size between 1 nm and 5 nm, as seen in Fig. 1c and d. This is in agreement with previous studies, in which the formation of Si clusters in a-SiN_x_ have been reported, detected using Raman scattering^40^, transmission electron microscopy (TEM), optical absorption and photoluminescence spectroscopy (PL)^41^.

Based on the determined composition, the mass density was determined by linear interpolation (equation (2)) to be 2.67 g/cm^3^. Using the bulk plasmon energy of the material, determined using electron energy loss spectroscopy (EELS) and equation (6) to be 20.6 eV, in equations (3), (4) and (5) with the composition measured by XPS and SIMS, and a band gap of 3 eV^42^, the mass density is estimated to be 2.43 g/cm^3^. The latter method employed on crystalline stoichiometric Si_3_N_4_ particles (Sigma Aldrich) resulted in a mass density of 3.24 g/cm^3^, which correspond well with the mass density of crystalline silicon nitride (3.18–3.19 g/cm^3^ ^43^). Given this and the wide variation of reported PECVD a-SiN_x_ densities in literature, a density of 2.43 g/cm^3^ is used in the specific capacity determination. Similarly, the mass density of the pure silicon reference was determined to be 2.18 g/cm^3^, using a band gap value of 1.9 eV^44^.

### Electrochemical testing

**Figure 2:**
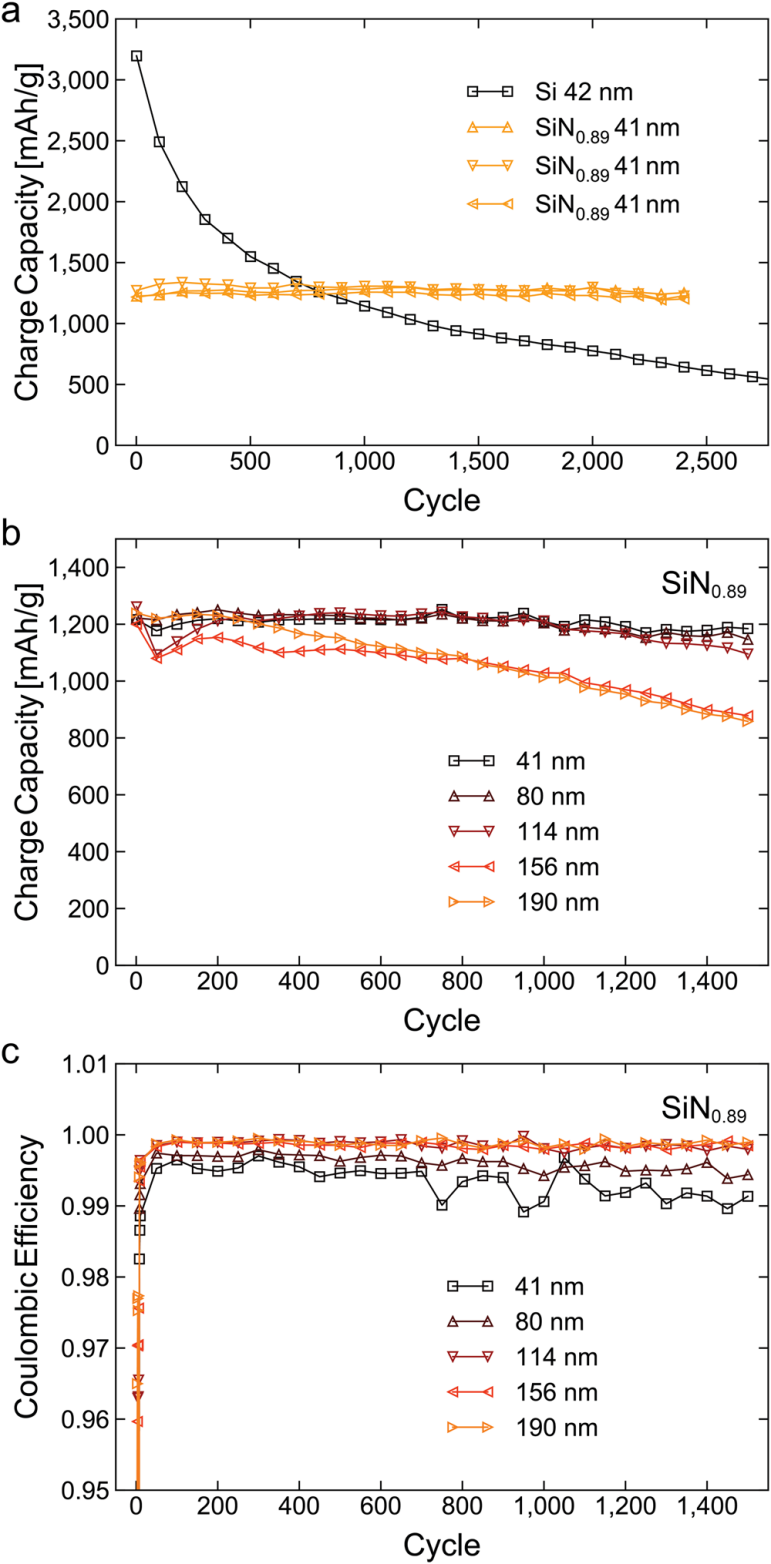
Charge capacity and Coulombic efficiency of different a-SiN_0.89_ thin films and a silicon reference. Charge capacity of three 41 nm a-SiN_0.89_ electrodes compared to a pure silicon reference cycled at C/3 under the same conditions (**a**). Charge capacity (**b**) and Coulombic efficiency (**c**) of a-SiN_0.89_ electrodes of different thicknesses cycled at 1 C.

The cycling performance of three electrodes of the 41 nm film cycled at C/3 is shown in Fig. 2a. From this, it is evident that the material not only is electrochemically active, but also exhibits high capacity and excellent cycling stability, with negligible capacity degradation over more than 2,400 cycles. The average charge capacity of the three a-SiN_0.89_ electrodes during the first 2,400 cycles was 1,266 ± 24 mAh/g. For comparison, Fig. 2a also shows the performance of a PECVD deposited amorphous silicon thin film of similar thickness, cycled under the same conditions. The starting capacity of this electrode is considerably higher than that of the nitride, but it suffers from heavy deterioration, and retains only 20.1% of its initial capacity after 2,400 cycles. While thin (<50 nm) silicon thin films have in some cases been demonstrated to exhibit good cycling stability^45^, particularly in all-solid-state batteries^24^, thicker films generally suffer from heavy cracking and delamination within some tens of cycles^46^. A comparison of the cycling performance of a-SiN_0.89_ electrodes of different thickness can be seen in Fig. 2b, which shows similar excellent cycling stability for the electrodes with thickness up to 114 nm. The 156 nm and 190 nm films, while showing some capacity degradation, still retain approximately 800 mAh/g after 1,500 cycles.

The average Coulombic efficiency and charge capacity over the first 1,000 cycles, and the average first cycle Coulombic efficiency for the different films can be seen in Table 1. To limit the effect of experimental variation, the values in this table are the calculated averages of two identical cells of each film type and thickness cycled in parallel. The low first cycle efficiency of all the nitrides is a result of a combination of lithium loss during SEI formation and the losses associated with the conversion reaction. The first cycle efficiency therefore is expected to increase with thickness as surface losses become of less importance, which was indeed observed. With regards to long term cycling, on the other hand, a reasonable assumption would be that thinner films have a higher Coulombic efficiency, given that thicker films generally are more prone to fracturing. As can be seen in Fig. 2c, this was not the case for the nitrides, which goes to show that fracturing is not a major issue in these electrodes, and that a majority of the long term lithium loss occurs at the surface. The oscillations in the Coulombic efficiency after 700 cycles were correlated to the ambient temperature during testing, with higher temperature giving slightly lower Coulombic efficiency.

**Table 1:** Key data from electrochemical testing of the different a-SiN_0.89_ thin films and a silicon reference.

Film Thickness [nm]	Mass loading [µg/cm^2^]	First cycle Coulombic efficiency	Average over 1,000 cycles			Capacity retention after 1,000 cycles
			Coulombic efficiency	Volumetric Charge Capacity [mAh/cm^3^]	Specific Charge capacity [mAh/g]	
41	10.0	30.8 ± 0.7%	99.4 ± 0.1%	2,804 ± 115	1,168 ± 47	99.5 ± 0.0%
80	19.3	43.1 ± 0.6%	99.6 ± 0.1%	2,924 ± 17	1,218 ± 7	99.5 ± 1.0%
114	27.8	50.1 ± 0.0%	99.8 ± 0.0%	2,996 ± 93	1,248 ± 38	95.3 ± 0.8%
156	37.8	52.2 ± 0.1%	99.8 ± 0.0%	2,507 ± 126	1,045 ± 52	85.5 ± 0.3%
190	46.2	54.6 ± 0.1%	99.8 ± 0.0%	2,690 ± 55	1,121 ± 23	82.1 ± 0.3%
42 (Si)	9.2	63.7 ± 0.1%	99.6 ± 0.0%	3,691 ± 88	1,678 ± 40	36.1 ± 0.3%

First cycle Coulombic efficiency, average Coulombic efficiency, volumetric charge capacity and specific charge capacity over the first 1,000 cycles, and capacity retention after 1000 cycles of the five different a-SiN_0.89_ films, as well as a 42 nm pure silicon thin film. All values are the average of two identical cells of each film type and thickness cycled in parallel.

**Figure 3:**
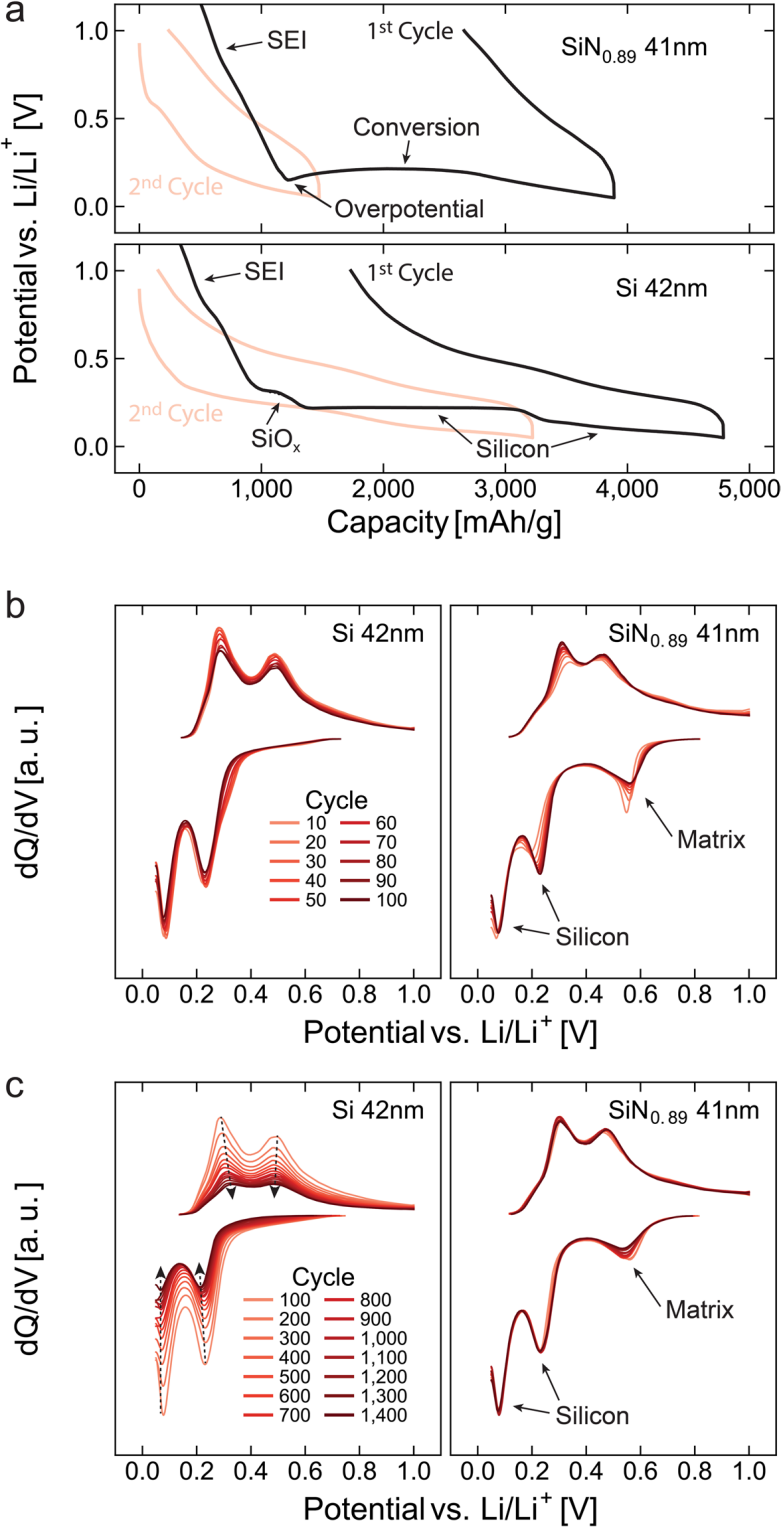
Comparison of the voltage-capacity and differential capacity plots of comparable a-SiN_0.89_ and silicon thin films. Voltage-capacity plots of the two initial lithiations/delithiations of a 41 nm a-SiN_0.89_ electrode and a 42 nm a-Si electrode (**a**), and differential capacity analysis of the same electrodes for cycles 10–100 (**b)**, and 100–1,400 (**c**).

Voltage-capacity plots focusing on the primary lithiation and delithiation of a 41 nm a-SiN_0.89_ and a 42 nm Si electrode can be seen in Fig. 3a. Examination of these plots largely confirms the expected differences between the nitride and silicon electrodes: For the silicon electrode, the two silicon lithiation plateaus begin after a typical oxide feature. The silicon nitride, on the other hand, lacks the typical silicon plateaus, but rather begins with a potential overshoot. This is characteristic of conversion reactions and corresponds to an overpotential related to the activation energy of nucleation of the new phase domains^32^. The kinetics of nucleation is typically limited, and it is noted that the initial cycle had to be run at a low C-rate in order to prevent the voltage minimum from reaching the cut-off at 50 mV before the conversion begins. This presents a possible explanation for the start-up problems reported for similar electrodes by Ahn, *et al*.^37^. In the subsequent lithiations, the conversion plateau at ~0.2 volts has mostly disappeared, showing that the conversion reaction happens largely to completion during the initial cycle and is irreversible.

Interestingly, during the next tens of cycles, the dQ/dV signature of the nitride begins to resemble that of the pure silicon film, as seen in Fig. 3b. This supports the hypothesis that the main active component of converted a-SiN_x_ is indeed silicon. There are two key differences, however; the presence of the extra discharge peak at 550 mV and increased activity at higher voltages during charge compared to pure a-Si. This is a strong indication that the matrix which forms during the conversion is, in fact, not completely inactive, but partially delithiates at higher potentials. It would then be reasonable to assume that the additional discharge peak is associated with the “relithiation” of this phase. While the magnitude of the matrix delithiation stays largely the same during the long term cycling, it is markedly higher during the initial slow cycles, suggesting that the delithiation is kinetically limited. The limited kinetics would then account for the sudden reduction and then stabilization of the “relithiation” peak during the first fast cycles - as the slow cycles end with a delithiation, the matrix is left with a lithium deficiency that is not fully compensated for until after several tens of fast cycles, when steady state at the new rate is established.

In the dQ/dV analysis of the next 1,400 cycles, which is seen in Fig. 3c, the capacity fade of the pure silicon electrode is clearly seen in the form of declining peak intensity. No significant peak shift is observed, however, indicating that the loss mechanism for the a-Si primarily is mechanical loss of active material, rather than chemical changes or increased cell resistance. Considering the small thickness of the film it is assumed that dimensional stabilization effectively reduces internal stress formation, and that the main mode of material loss therefore is delamination. During the same cycles the nitride continues to exhibit remarkable stability, both with regards to peak intensity and position. This stability is, however, also observed to be largely sustained for the thicker films, where internal stress formation typically begins to rapidly degrade silicon anodes. As the capacity of the nitride is lower than for silicon, the volume change during cycling is expected to be substantially smaller. This can account for both the reduced delamination and reduced internal stress formation. In practice, the nitride behaves as a *self-limiting* silicon anode material. Silicon migration during cycling has also been reported as detrimental to dimensional stabilization – coarsening otherwise nanostructured anodes^4^. This effect is expected to be reduced in the a-SiN_x_ electrodes due to restrictions imposed on the migration of the nano-silicon domains by the matrix phase.

The previous reports on the electrochemical performance of silicon nitride in lithium ion batteries have shown widely varying results from a variety of systems: Crystalline stoichiometric Si_3_N_4_ is generally reported to exhibit negligible capacity^35,38^, which may be a reason for the limited attention silicon nitride has received in the context of lithium ion batteries. The few investigations on amorphous silicon nitrides, on the other hand, generally report at least some reversible capacity: Ahn, *et al*.^37^ reported that a silicon rich thin film electrode, SiN_0.32_, exhibited a capacity of 2,300 mAh/g over 200 cycles^37^. This happened after several hundred cycles with negligible capacity, which we suspect is related to the previously addressed potential overshoot at the beginning of the conversion. Most comparable to the experiments conducted in this work are those published by Suzuki, *et al*.^36^, who reported largely similar performance from a-SiN_0.92_ thin films over 100 cycles as was observed here. Beyond the few hundred cycles in these reports, however, to the authors’ knowledge, there is no precedence for the long term cycling performed in this work. The high capacity and excellent cycling stability exhibited during these tests emphasizes the potential of amorphous silicon nitride as a new generation anode material.

## Conclusions

Electrochemical testing has shown that a-SiN_0.89_ has a specific capacity of approximately 1,200 mAh/g, which, while considerably lower than silicon, is still more than three times the capacity of current carbon electrodes, and at a level where further improvement has limited effect on the full cell capacity^2^. The material also exhibits excellent cycling stability, particularly the thinnest films, which retain a capacity of 1,266 ± 24 mAh/g after 2,400 cycles. The material does, however, exhibit a low first cycle Coulombic efficiency caused by lithium lost to the matrix formation during the conversion reaction. Low initial Coulombic efficiency is a common issue for high capacity anode materials, and underlines the importance of developing an efficient prelithiation method.

Using differential capacity analysis it was shown that the primary active component of the converted a-SiN_0.89_ electrode is indeed silicon. However, this analysis also revealed some additional activity at higher voltages, which has been interpreted as the matrix not being completely inactive, but rather being partially delithiated and “relithiated”. An increase in this activity at lower C-rate indicates that this process is primarily kinetically limited.

We believe that the high capacity and excellent cycling performance of this material warrants further research. An investigation of the effect of composition on the performance of a-SiN_x_ anodes is already in progress, and the results will be the subject of a forthcoming publication. We expect that if powder based a-SiN_x_ electrodes can be demonstrated to exhibit similar performance, this may be of substantial value for commercial applications.

## Methods

### Film deposition

Thin films were deposited using PECVD (Oxford Instruments Plasmalab System133) with silane (SiH_4_) and ammonia (NH_3_) as precursors. The a-SiN_x_ films were deposited with a plasma power of 40 W on copper substrates which had a temperature of 400 °C. The chamber pressure was kept at 300 mTorr and the flow rates of SiH_4_ and NH_3_ were 25 sccm and 40 sccm, respectively. Using deposition times of 160 s, 320 s, 480 s, 640 s, and 800 s, this typically results in film thicknesses of 40 nm, 80 nm, 120 nm, 160 nm, and 200 nm. A pure silicon reference was made without NH_3_, but with otherwise the same process parameters, and a deposition time of 160 s.

### Ellipsometry

The film thicknesses and refractive indices were measured using spectroscopic ellipsometry (V-VASE® J.A. Woollam Co.). This analysis requires a flat surface, and was therefore conducted on films deposited on polished silicon wafers, which were put in the deposition chamber together with the copper substrates.

### Electron and Optical Microscopy

Selected films were investigated using TEM. The instrument used for this characterization was a probe corrected FEI Titan G2 60–300 operated at 300 kV in scanning TEM mode (STEM). Cross section TEM samples were prepared by focused ion beam (FIB) milling on a JEOL JIB-4500 dual beam system. The surface morphology and coverage of the films were characterized using optical microscopy and scanning electron microscopy (SEM, Hitachi TM3000 & JEOL JIB-4500).

### X-ray Photoelectron Spectroscopy (XPS)

XPS analysis was conducted on a Kratos Axis Ultra DLD instrument using monochromated Al Kα X-rays (hν = 1,486.6 eV). In order to remove any surface contamination, the top surface of the films was removed by argon sputtering at 2 kV and 100 µA for 2 minutes before characterization. Elemental quantification was done based on O 1 s, N 1 s, and Si 2p spectra after Shirley background subtraction^47^. Peak fitting of XPS spectra was done using a procedure from Ingo, *et al*.^39^.

### Secondary Ion Mass Spectrometry (SIMS)

SIMS analysis was conducted to measure the hydrogen content of the thin films. This was done using a Cameca IMS 7 f double-focusing magnetic sector spectrometer, using a cesium primary ion beam with impact energy of 15 kV. The best available quantification reference was a standard sample of hydrogen implanted single crystalline silicon, which was used to determine the relative sensitivity factors for silicon and hydrogen. Matrix effects caused by the nitrogen content and amorphicity of the a-SiN_x_ thin films are therefore not accounted for, and the result of the analysis should therefore be regarded as a rough estimate of the hydrogen content.

### Composition Determination

To determine the film compositions, different methods were used in conjunction. A commonly used method is to estimate the composition from the refractive index using a formula proposed by Bustarret, *et al*.^48^.

1 $$\frac{[N]}{[Si]}=\frac{4}{3}\frac{({n}_{Si}-{n}_{film})}{({n}_{film}+{n}_{Si}-2{n}_{S{i}_{3}{N}_{4}})}$$

Here *n*_*Si*_, *n*_*Si3N4*_ and *n*_*film*_ are the refractive indices of amorphous silicon, silicon nitride and the film, respectively. The values used for the refractive indices at λ = 630 nm of silicon and silicon nitride are 4.2115^49^ and 2.0108^50^, respectively. The accuracy of this formula is dependent on, among other factors, the hydrogen content of the film, and is therefore dependent on the fabrication parameters. The applicability of equation (1) in these experiments is evaluated by comparison with compositions estimated from the XPS measurements.

### Film Density Determination

The mass density of the films, which was used in calculating the specific capacities of the material, was estimated using two methods. The first method estimates the film density from the composition by linear interpolation based on the N/Si atomic ratio between the reported density of pure amorphous silicon and a-SiN_x_ in literature. The reference values used for this purpose were *ρ*_*Si*_ = 2.2 g/cm^3^ for a-Si^51^ and *ρ*_*SiN1.31*_ = 2.9 g/cm^3^ for a-SiN_1.31_^52^, resulting in

2 $${\rho }_{l}={\rho }_{Si}+({\rho }_{Si{N}_{1.31}}-{\rho }_{Si})\frac{{f}_{N}}{1.31(1-{f}_{N})}$$

Here *ρ*_*l*_ is the linearly interpolated mass density, and *f*_*N*_ is the molar fraction of nitrogen in the film. As the reported mass densities of a-Si and a-SiN_x_ vary significantly, a second method was also used, in which the density of the films is determined using the characteristic bulk plasmon energy as measured in the TEM using EELS. This method is based on the free electron model, in which the bulk plasmon energy can be related to the valence electron density by the formula

3 $${E}_{p}=\hslash \sqrt{\frac{n{e}^{2}}{m{{\epsilon }}_{0}}}$$

Here *E*_*p*_ is the bulk plasmon energy, *ħ* is the reduced Planck constant, *n* is the valence electron density, *e* is the elementary charge, *m* is the electron mass and *ε*_0_ is the permittivity of vacuum. Since the free-electron model is primarily expected to apply to metals, a correction can be made for non-metals by using the band gap of the material as a measure of the electron binding energy. Its impact on the plasmon energy is formulated by Egerton^53^ as

4 $${E}_{p}=\sqrt{{{E}_{p,bound}}^{2}-{{E}_{g}}^{2}}$$

Here *E*_*p*_ and *E*_*p, bound*_ are the plasmon energies in systems with the same valence electron density with free and bound electrons, respectively, and *E*_*g*_ is the band gap of the material. Assuming that each hydrogen, silicon, nitrogen, oxygen atom has 1, 4, 5, and 6 valence electrons, respectively, the valence electron density can then be related to the mass density, *ρ*_*p*_, by the formula

5 $${\rho }_{p}=n\ast \frac{{f}_{Si}{M}_{Si}+{f}_{N}{M}_{N}+{f}_{O}{M}_{O}+{f}_{H}{M}_{H}}{{N}_{A}(4{f}_{Si}+5{f}_{N}+6{f}_{O}+1{f}_{H})}$$

Here *N*_*A*_ is the Avogadro constant, and *f*_*X*_ and *M*_*X*_ are the molar fraction and molar mass of element X, respectively. The plasmon energy, *E*_*p, bound*_, is extracted from the EELS-spectra using a formula by Egerton^53^

6 $${E}_{p,bound}=\sqrt{{{E}_{max}}^{2}+\frac{{\rm{\Delta }}{{E}_{p}}^{2}}{2}}$$

Here *E*_*max*_ is the energy of the observed plasmon peak maximum and *ΔE*_*p*_ is the full width at half maximum of the plasmon peak.

### Electrochemical Testing

For electrochemical testing, Ø15 mm discs were punched from the deposited films and mounted in 2032 coin cells against a lithium metal counter electrode, with a Celgard 3401 separator and using an electrolyte consisting of 1 M LiPF_6_ in ethylene carbonate (EC)/propylene carbonate (PC)/dimethyl carbonate (DMC) (1:1:3 by mass), with 1 wt.% vinylene carbonate (VC) and 5 wt.% fluoroethylene carbonate (FEC). The cells were cycled between 0.05 V and 1 V using an Arbin BT-2000 galvanostat/potentiostat. The initial cycle, during which the conversion happens, was performed at a rate of C/20. To ensure that full conversion is achieved, the cells were cycled at C/20 for 5 additional cycles before long term cycling was performed at either C/3 or 1 C. A C-rate of 1 C = 1,200 mA/g was used for all a-SiN_x_ thin films, which was based on the specific capacity determined from preliminary cycling tests of the 41 nm a-SiN_x_ film. For the pure silicon thin film a rate of 1 C = 3,579 mA/g was used, corresponding to the theoretical capacity of silicon. The mass of the electrodes used to determine the total current used during cycling was calculated from the film thickness and density. The former as measured by ellipsometry and the latter as determined from the bulk plasmon energy, which was measured using EELS.

## Electronic supplementary material

Supplementary Information
