# Supplementary material for: Long-term Cyclability of Substoichiometric Silicon Nitride Thin Film Anodes for Li-ion Batteries
Source: Sci Rep. 2017 Oct 17;7:13315. doi: 10.1038/s41598-017-13699-0 (PMC5645470; doi:10.1038/s41598-017-13699-0)
Supplement: Supplementary file 1 — Supplementary Information [file 41598_2017_13699_MOESM1_ESM.pdf]

# Supplementary information to the paper:

# Long-term Cyclability of Substoichiometric Silicon Nitride Thin Film Anodes for Li-ion Batteries

**Asbjørn Ulvestad[a, b], Hanne Flåten Andersen[a], Jan Petter Mæhlen[a], Øystein Prytz[b], and Martin Kirkengen[a, b]**

[a] Department of Energy Systems, Institute for Energy Technology
P. O. Box 40, NO-2027 Kjeller, Norway

[b] Department of Physics, Centre for Materials Science and Nanotechnology, University of Oslo
P. O. Box 1048 Blindern, NO-0316 Oslo, Norway

# 1 Electrochemical cycling parameters

## 1.1 Rate determination

As the conversion reaction of silicon nitrides has not been conclusively determined, the material's theoretical capacity cannot be directly calculated. Because of this, preliminary tests using 41 nm $SiN_{0.89}$ thin film electrodes have been conducted in order to find an approximate theoretical capacity of the material. Three electrodes were cycled with a current density of 2.0 µA/cm$^2$ for 5 cycles to allow the conversion reaction to happen fully. The electrodes were then cycled at 13.5 µA/cm$^2$ for 50 cycles. The average capacity of these three electrodes during the final 50 cycles was 1213 ± 39 mAh/g, which was rounded to 1200 mAh/g. Based on this, the current densities used in the preliminary tests correspond to C-rates of approximately C/6 for the conversion cycles and 1.125C for the subsequent cycles, which we assume to be sufficiently slow to reflect the full capacity of the cell.

Based on this capacity, the absolute current used for each electrode was determined using the mass of the electrodes, as calculated from the film thickness and density. The current rate for the silicon reference was determined using the theoretical capacity of silicon and corresponding C-rate (3579 mA/g). The parameters used in the calculation are shown in Supplementary Table 1 and the resulting current densities and rates are shown in Supplementary Table 2.

**Supplementary Table 1: Overview of the different films and the parameters used in determining the current rates used during cycle testing: The thickness, density and mass loading of the films, the assumed specific capacity, and the calculated areal capacity.**

| Film material | Film thickness [nm] | Density [g/cm$^2$] | Mass loading [µg/cm$^2$] | Specific capacity [mAh/g] | Areal capacity [µAh/cm$^2$] |
|---|---|---|---|---|---|
| a-SiN0.89 | 41 | 2.43 | 10.0 | 1200 | 12.0 |
| a-SiN0.89 | 80 | 2.43 | 19.3 | 1200 | 23.3 |
| a-SiN0.89 | 114 | 2.43 | 27.8 | 1200 | 33.2 |
| a-SiN0.89 | 156 | 2.43 | 37.8 | 1200 | 45.5 |
| a-SiN0.89 | 190 | 2.43 | 46.2 | 1200 | 55.4 |
| a-Si | 42 | 2.18 | 9.2 | 3579 | 32.8 |

**Supplementary Table 2: Overview of the current densities used during cycling of the different films at different C-rates, and the absolute currents used for cycling of ⌀15 mm electrodes of each film.**

| Film material | Film thickness [nm] | Current density [µA/cm$^2$] | | | Assumed capacity of a ⌀15 mm electrode [µAh] | Current rate for a ⌀15 mm electrode [µA] | | |
|---|---|---|---|---|---|---|---|---|
| | | C/20 | C/3 | 1C | | C/20 | C/3 | 1C |
| a-SiN0.89 | 41 | 0.60 | 3.99 | 12.0 | 21.1 | 1.06 | 7.04 | 21.1 |
| a-SiN0.89 | 80 | 1.17 | 7.78 | 23.3 | 41.2 | 2.06 | 13.7 | 41.2 |
| a-SiN0.89 | 114 | 1.66 | 11.1 | 33.2 | 58.7 | 2.94 | 19.6 | 58.7 |
| a-SiN0.89 | 156 | 2.27 | 15.2 | 45.5 | 80.4 | 4.02 | 26.8 | 80.4 |
| a-SiN0.89 | 190 | 2.77 | 18.5 | 55.4 | 97.9 | 4.90 | 32.6 | 97.9 |
| a-Si | 42 | 1.64 | 10.92 | 32.77 | 57.9 | 2.90 | 19.3 | 57.9 |

## 1.2 Galvanostatic Voltage-Capacity Curves

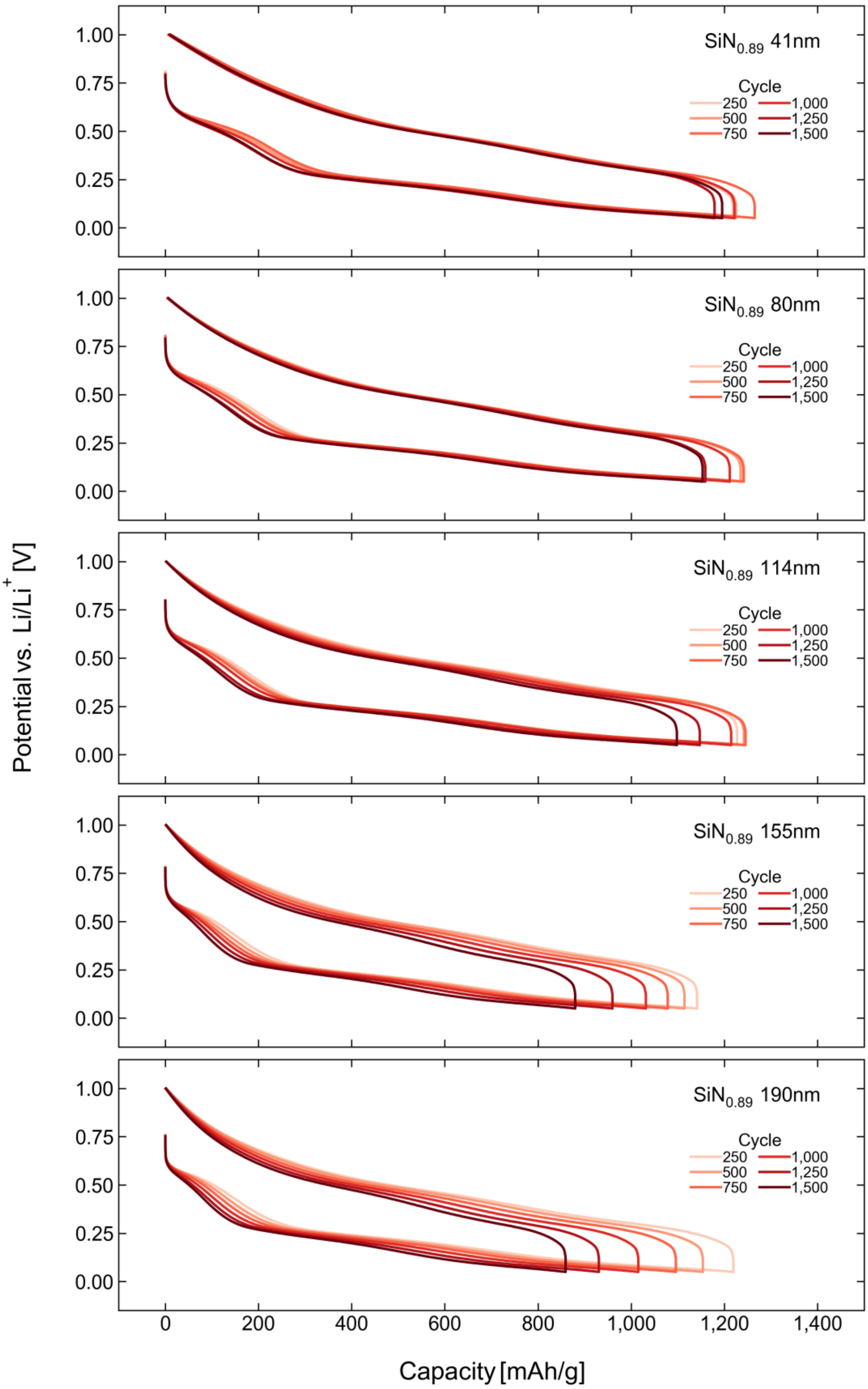


**Supplementary Figure 1: Galvanostatic voltage-capacity curves from cycles 250, 500, 750, 1000, 1250, and 1500 of five $SiN_{0.89}$ thin film electrodes with thickness 41, 80, 114, 156 and 190 nm, cycled at 1C.**

# 2 X-ray Photoelectron Spectroscopy

## 2.1 Si 2p peak fitting

By fitting Voigt functions to the Si 2p core level XPS spectra acquired from the 41 nm and 114 nm $SiN_{0.89}$ thin films using a procedure by Ingo, et al. [39], the distribution of silicon atoms in Si-$Si_4$ (pure Si), Si-$Si_3$N ($Si_3$N), Si-$Si_2N_2$ ($Si_3N_2$), Si-$SiN_3$ ($Si_3N_3$), and Si-$N_4$ ($Si_3N_4$) configuration was determined. This fitting can be seen in Supplementary Figure 2, and the resulting distribution can be seen in the next section.

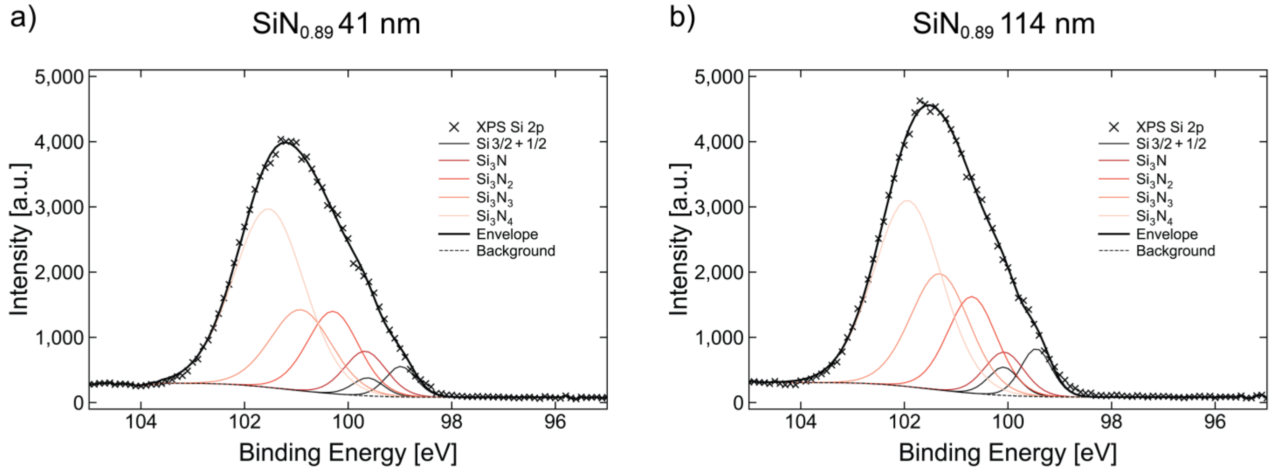


**Supplementary Figure 2: Fitting of Voigt functions to Si 2p core level XPS spectra obtained from the 41 nm and 114 nm $SiN_{0.89}$ thin films.**

## 2.2 Silicon coordination distribution fitting

A comparison of the measured silicon coordination distribution with a calculated distribution for homogenous $SiN_x$ using a random mixing model (RMM) can be seen in Supplementary Figure 3a. The Si-$SiN_3$ ($Si_3N_3$) and Si-$N_4$ ($Si_3N_4$) components are combined in order to reduce the reliance on component separation and prevent overfitting. This shows some disagreement between the measurement and model, notably that the number of silicon atoms in a pure Si configuration, which should be close to zero, is higher than expected, indicating that some precipitation of pure silicon has occurred. This is in agreement with TEM analysis, in which some phase separation was observed. The precipitation of Si would necessarily lead to an enrichment of nitrogen in the remaining $SiN_x$. Taking the initial Si coordination distribution and subtracting the amount of pure Si component that has precipitated would, after normalization, effectively describe the corresponding distribution in the enriched $SiN_x$. Using the amount of pure Si precipitated as a fitting parameter, the composition and Si coordination distribution of the enriched $SiN_x$ was fitted to the calculated distribution (RMM), the result of which can be seen in Supplementary Figure 3b. This shows good agreement between the measured and calculated distributions, supporting the initial hypothesis that some pure Si has indeed precipitated.

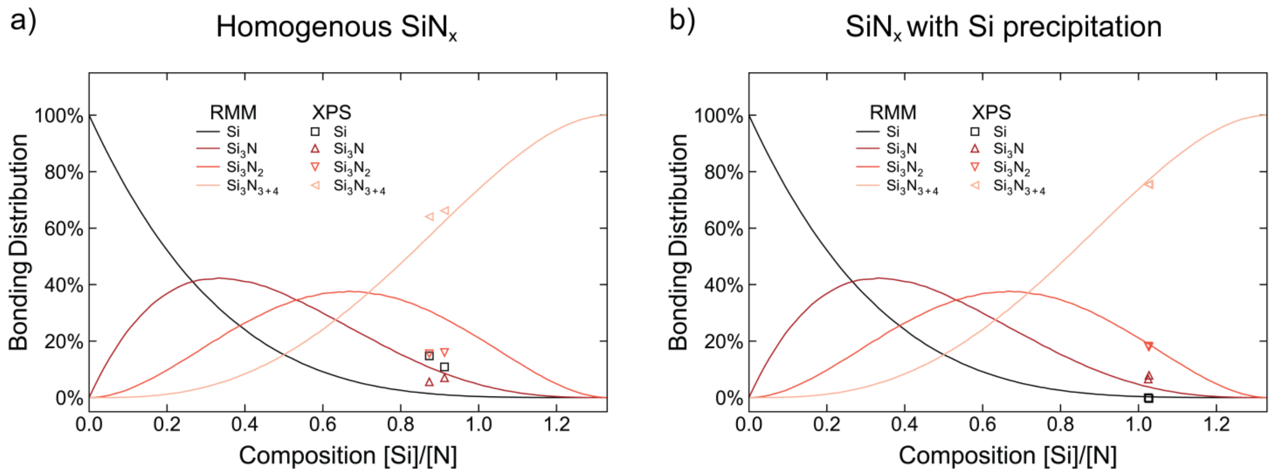


**Supplementary Figure 3: Silicon coordination distribution as measured using XPS (markers) and calculated (lines) using a random mixing model (RMM).** a) Assuming homogenous $SiN_x$, and b) assuming some precipitation of pure Si. Subtracting that from the initial distribution and normalizing results in the distribution of Si configurations in the nitrogen enriched $SiN_x$, which, after normalization, is fitted to the calculated distribution using the amount of pure silicon precipitated as a fitting parameter.

# 3 Ellipsometry

Ellipsometry measurements of the five $SiN_{0.89}$ films were acquired using wavelengths from 600 to 1000 nm and five angles of incidence: 60, 65, 70, 75, and 80 degrees. Assuming that the films are transparent in this range of wavelengths, the refractive index and thickness of each thin film was determined by fitting a Cauchy equation to the ellipsometry data. The fits can be seen in Supplementary Figures 4 to 8, showing good agreement between model and measured data, and the resulting parameters are shown in Supplementary Table 3.

**Supplementary Table 3: The thickness and refractive index of the films as determined from Cauchy model fitting to ellipsometry data, seen in Supplementary Figures 4 to 8.**

| Deposition time [s] | Film thickness [nm] | Refractive index (λ=630 nm) |
|---|---|---|
| 160 | 41 | 2.24 |
| 320 | 80 | 2.35 |
| 480 | 114 | 2.37 |
| 640 | 156 | 2.38 |
| 800 | 190 | 2.27 |

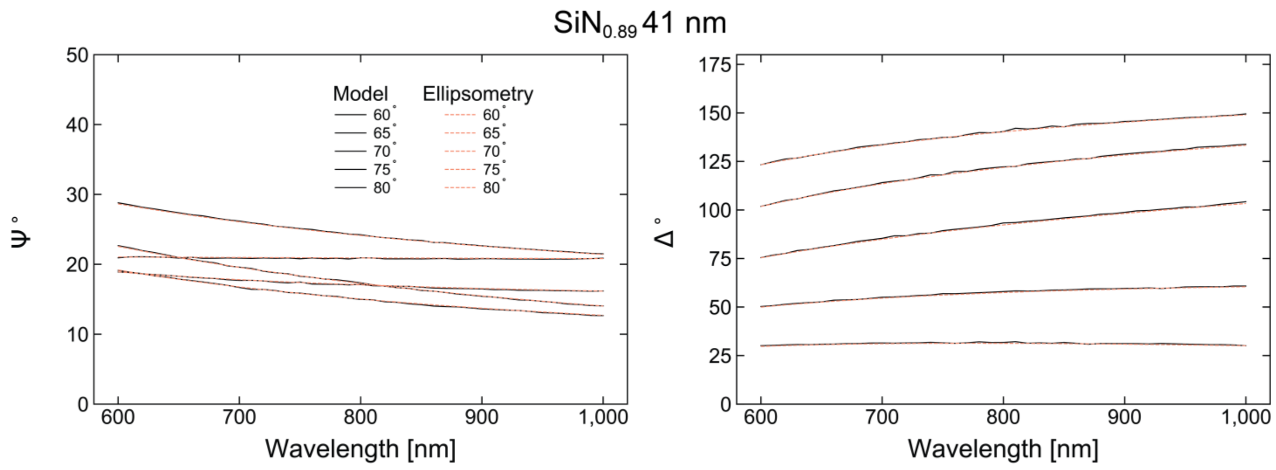


**Supplementary Figure 4: Ellipsometry measurement and Cauchy model fit for the 41 nm $SiN_{0.89}$ film.**

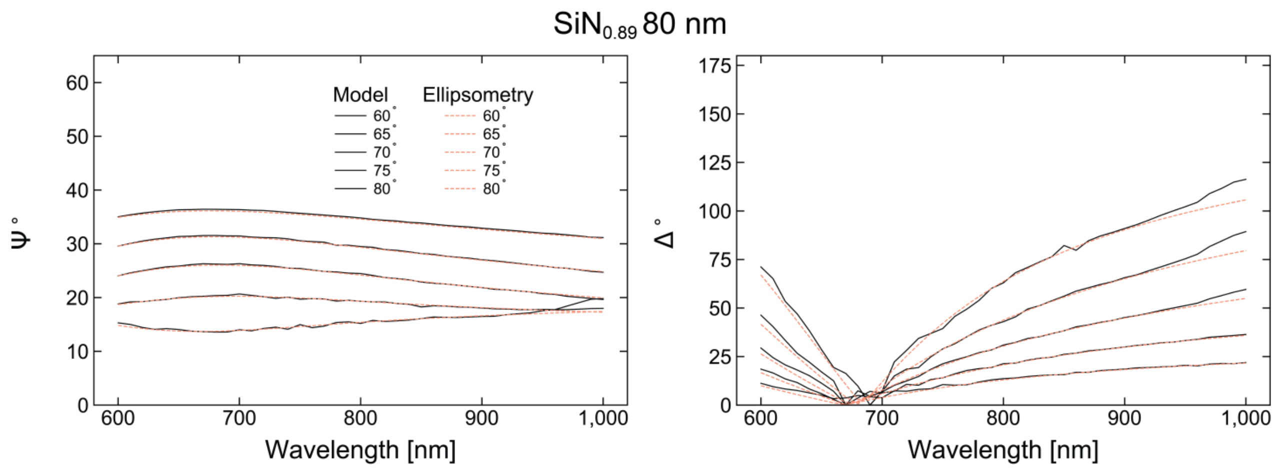


**Supplementary Figure 5: Ellipsometry measurement and Cauchy model fit for the 80 nm $SiN_{0.89}$ film.**

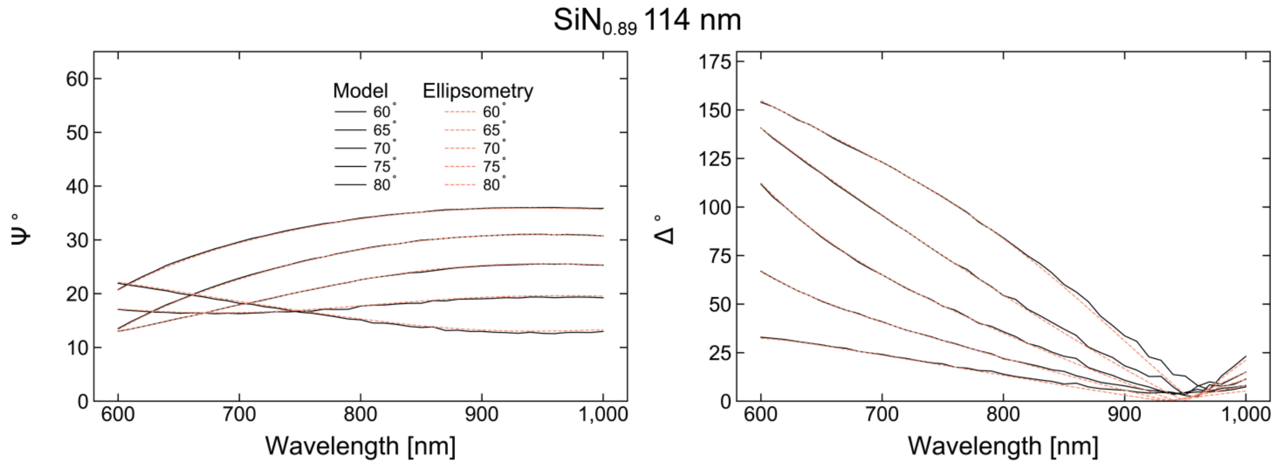


**Supplementary Figure 6: Ellipsometry measurement and Cauchy model fit for the 114 nm $SiN_{0.89}$ film.**

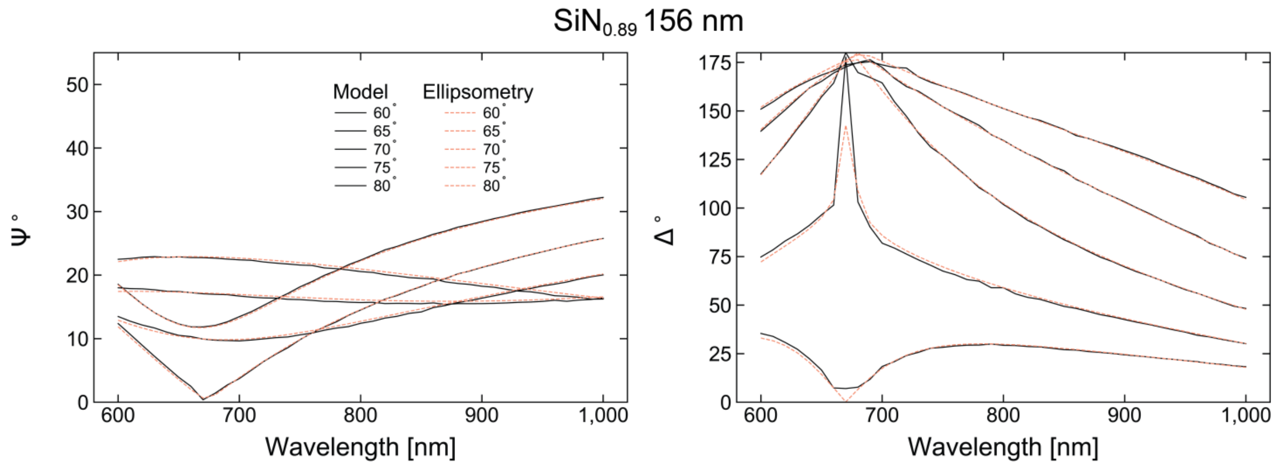


**Supplementary Figure 7: Ellipsometry measurement and Cauchy model fit for the 156 nm $SiN_{0.89}$ film.**

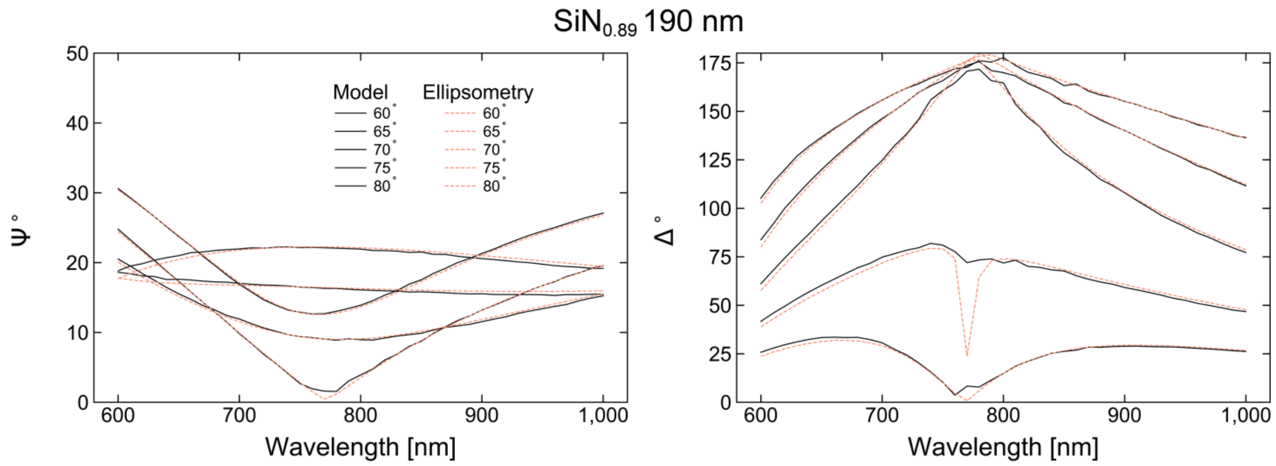


**Supplementary Figure 8: Ellipsometry measurement and Cauchy model fit for the 190 nm $SiN_{0.89}$ film.**

# 4 Density determination

## 4.1 Bulk plasmon energy determination from electron energy loss spectroscopy (EELS)

The bulk plasmon energy of the $SiN_{0.89}$ was determined using EELS analysis of the 114 nm thin film. This analysis was conducted in an FEI Titan G2 60-300 TEM operating at 300 kV on a cross-section TEM sample prepared using a JEOL JIB-4500 focused ion beam system. Spectra were aquired using a Gatan GIF Quantum 965 EELS spectrometer at a dispersion of 0.025 eV/channel. A total of 936 spectra were aquired over the thickness of the film, aligned and summed, resulting in the raw spectrum seen in Supplementary Figure 9. To remove plural scattering contributions, the raw spectrum was deconvoluted using the Fourier-log method. The resulting deconvoluted spectrum, also seen in the same figure, was used to extract the bulk plasmon peak maximum and full width at half maximum (FWHM), which was found to be 20.0 eV and 10.7 eV, respectively. Based on these values and equation (6) in the main paper, the bulk plasmon energy was determined to be 21.4 eV.

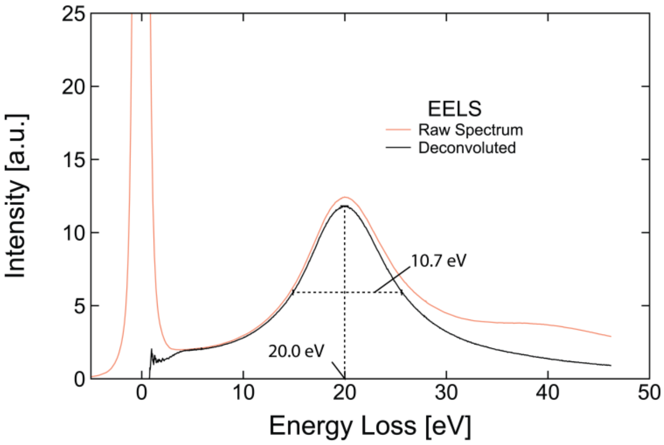


**Supplementary Figure 9: EELS spectrum acquired from the 114 nm $SiN_{0.89}$ thin film, both as-recorded and deconvoluted using the Fourier-log method. The position of the bulk plasmon peak maximum and FWHM are indicated.**

## 4.2 Calculation of mass density from bulk plasmon energy

The density of the deposited $SiN_{0.89}$ was determined from the bulk plasmon energy by the procedure outlined in the methods section in the main paper. The stages of the calculation are shown in Supplementary Table 4. Firstly, a free electron environment equivalent of the bulk plasmon energy is calculated from the measured *bound* bulk plasmon energy using equation (4) and a band gap value of 3 eV [(42)]. This value is then used to estimate the valence electron density using equation (3), which is then related to the mass density of the material through equation (5). In this calculation, the hydrogen content of the film was assumed to be 20 at. %, based on an estimate obtained from secondary ion mass spectrometry (SIMS) analysis. The content of silicon, nitrogen and oxygen in the material was determined by XPS to be 40.7 at. %, 35.6 at. % and 3.7 at. %, respectively. This resulted in a mass density of 2.43 g/cm$^3$. For similar analysis of the pure silicon reference, a band gap of 1.9 eV [(44)], an oxygen content of 3.9 at. % as measured using XPS, and the same estimated hydrogen content as the nitride films resulted in a mass density of 2.18 g/cm$^3$.

**Supplementary Table 4: Density determination from bound bulk plasmon energy to mass density using equations (3) to (5) from the main paper.**

| **Stage** | **$SiN_{0.89}$** | **Pure Si reference** |
|---|---|---|
| Bound bulk plasmon energy from EELS | 21.4 eV | 17.0 eV |
| Equation (4) -> Free bulk plasmon energy | 21.2 eV | 16.9 eV |
| Equation (3) -> Valence electron density | $3.25*10^{29}$ cm$^{-3}$ | $2.09*10^{29}$ cm$^{-3}$ |
| Equation (5) -> Mass density | 2.43 g/cm$^3$ | 2.18 g/cm$^3$ |
